# Deciphering the Molecular Recognition Mechanism of Multidrug Resistance *Staphylococcus aureus* NorA Efflux Pump Using a Supervised Molecular Dynamics Approach

**DOI:** 10.3390/ijms20164041

**Published:** 2019-08-19

**Authors:** Deborah Palazzotti, Maicol Bissaro, Giovanni Bolcato, Andrea Astolfi, Tommaso Felicetti, Stefano Sabatini, Mattia Sturlese, Violetta Cecchetti, Maria Letizia Barreca, Stefano Moro

**Affiliations:** 1 Molecular Modeling Section (MMS), Department of Pharmaceutical and Pharmacological Sciences, University of Padova, via Marzolo 5, 35131 Padova, Italy; 2 Department of Pharmaceutical Sciences, “Department of excellence 2018-2022”, University of Perugia, Via del Liceo 1, 06123 Perugia, Italy

**Keywords:** antimicrobial resistance, norA efflux pump, homology modeling, molecular dynamics simulation, supervised molecular dynamics

## Abstract

The use and misuse of antibiotics has resulted in critical conditions for drug-resistant bacteria emergency, accelerating the development of antimicrobial resistance (AMR). In this context, the co-administration of an antibiotic with a compound able to restore sufficient antibacterial activity may be a successful strategy. In particular, the identification of efflux pump inhibitors (EPIs) holds promise for new antibiotic resistance breakers (ARBs). Indeed, bacterial efflux pumps have a key role in AMR development; for instance, NorA efflux pump contributes to *Staphylococcus aureus* (*S. aureus*) resistance against fluoroquinolone antibiotics (e.g., ciprofloxacin) by promoting their active extrusion from the cells. Even though NorA efflux pump is known to be a potential target for EPIs development, the absence of structural information about this protein and the little knowledge available on its mechanism of action have strongly hampered rational drug discovery efforts in this area. In the present work, we investigated at the molecular level the substrate recognition pathway of NorA through a Supervised Molecular Dynamics (SuMD) approach, using a NorA homology model. Specific amino acids were identified as playing a key role in the efflux pump-mediated extrusion of its substrate, paving the way for a deeper understanding of both the mechanisms of action and the inhibition of such efflux pumps.

## 1. Introduction

Antimicrobial resistance (AMR) is a complex global health challenge, mainly resulting from the excessive use and abuse of antimicrobial agents in humans and animals. [1] Indeed, over the years, the microbial world has developed the molecular tools to drive resistance and evade antibiotic action via (i) alteration of targeted site, (ii) enzymatic drug inactivation/modification, (iii) decreased uptake or enhanced efflux of the drug, and (iv) biofilm formation [2].

In this context, *Staphylococcus aureus* represents the most dangerous superbug among Gram-positive organisms due to its ability to develop resistance to a wide range of compounds [3]. *S. aureus* possesses several efflux pumps belonging to different families able to extrude a wide array of common antibacterial drugs [4]. NorA is a multidrug resistance (MDR) efflux pump, well-studied since 1986 when it was isolated from the urine of a patient treated with norfloxacin (NOR).

NorA was thus the first chromosomally-encoded *S. aureus* MDR pump to be identified: it is codified by norA gene and expressed in 43% of bacterial strains [5]. From a structural point of view, NorA is a single-chain transmembrane protein of 42,385 kDa composed of 388 amino acids. It belongs to the Major Facilitator Superfamily (MFS) consisting of 12 hydrophobic transmembrane (TM) α-helices with the N- and C-terminal domains that are placed in the cytoplasmic side, connected by hydrophilic loops and arranged as pseudo-twofold symmetry [6,7]. Unfortunately, little is known about the mechanism of efflux, except that it works by using the proton that allows the entry of a proton-coupled to the extrusion of the drug from the cell. Indeed, NorA is classified as a drug/H^+^ antiporter.

NorA overexpression is associated with drug resistance. In particular, NorA is a promiscuous efflux pump involved in quinolones and fluoroquinolones (such as ciprofloxacin—CPX) resistance [8], but also in the extrusion of other natural and synthetic structurally unrelated compounds (e.g., quaternary ammonium compounds and antiseptics, phenothiazines and thioxanthenes, totarol, ferruginol, carnosic acid, ethidium bromide (EtBr), tetraphenylphosphonium, rhodamine, acridine, and biocides) [8].

To date, several scientific efforts have been made to identify efflux pump inhibitors (EPIs) with the final aim to counteract the *S. aureus* resistance mechanism and restore bacterial susceptibility to antibiotic action [8,9,10,11,12,13,14,15,16].

Even though structure information of different drug/H^+^ antiporter are publicly available [17,18,19,20] in the RCSB Protein Data Bank (PDB) [21], unfortunately, neither 3D structures of NorA have been made public nor computational studies have been reported to understand the recognition mechanism between the efflux pump and the substrate.

Against this backdrop, the aim of the present work was to explore at the molecular level the possible recognition pathway and interactions between NorA efflux pump and its substrate CPX by using a Supervised Molecular Dynamics approach (SuMD) [22]. In brief, a SuMD simulation is composed of a number of consecutive short unbiased MD simulations (600 ps) in which a supervision strategy, based on a tabu search-like strategy, is applied at the end of each simulation. The supervised variable is the distance between the ligand and protein binding site center of mass (dcm _(L-R)_). In few words, if this distance is likely to be shortened during the simulation, the MD simulation is prolonged, otherwise, it is stopped, and the simulation is restarted from the previous set of coordinates. The supervision is maintained until the protein-ligand distance reaches a pre-set threshold value, then the simulation proceeds as a conventional unbiased MD simulation.

SuMD aided for the first time the recognition pathway of the efflux pump NorA, with the substrate CPX giving interesting information about the sites explored during its trajectory prior to extrusion toward the periplasmatic side.

## 2. Results and Discussion

### 2.1. Prediction and Assessment of the NorA 3D Structure

First, four bioinformatics tools—I-TASSER [23,24], SWISS-MODEL [25], RaptorX [26] and Phyre2 [27]—were used to generate NorA efflux pump homology models (Table S1; Supplementary Materials). Overall, two different conformations of NorA were obtained as output: an outward conformation (C_out_) with an opening toward the periplasmic side, and an inward conformation (C_in_) with an opening toward the cytoplasmic side. Given our main interest in the molecular recognition mechanisms underneath the interactions between a substrate and the transporter immediately antecedent to its extrusion, we decided to focus our subsequent studies on the predicted inward conformations. The different software used provided us with three C_in_ models using three different templates. Indeed I-TASSER, RaptorX, and Phyre2 produced C_in_ models built based on the MSF *E. coli* MdfA transporter (PDB: 4ZOW) [19], the MFS proton-dependent oligopeptide transporters (POTs) of *E. coli* (PDB: 4IKV) [28] and the MSF *E. coli* MdfA transporter (PDB ID 4ZP0), respectively (Table S1; Supplementary Materials). Interestingly, in the 4ZOW crystal structure, the MdfA efflux pump is co-crystallized with its substrate chloramphenicol (CLM).

The quality of the models was assessed on the basis of the geometry using MOE suite [29] and the Qualitative Model Energy ANalysis (QMEAN) value (Table S1). Model’s evaluation was also performed according to the Root Mean Square Deviations (RMSD). Noteworthy the RaptorX model RMSD was 3.234 Å, while the I-TASSER C_in_ RMSD on the template was 1.7 Å (Figure S1) and Phyre2 C_in_ RMSD was 0.35 Å. All models were good and reliable from a geometric point of view, quality of prediction and RMSD compared to the templates. To support the choice of our model we also carried out a sequence alignment with MOE suite to evaluate the sequence similarity and identity between the template and the NorA model generated. Although the percentage of sequence identity was low for all models, the choice of the model generated from MdfA was strongly supported by similarity (Figure S2).

We chose the I-TASSER model C_in_ because, at the same quality, it was built on a crystal in which the substrate was present [19].

### 2.2. MdfA Template and NorA Comparison

Aside from predicting homology models, the Phyre2 web portal provided useful information to better understand the evolutionary correlation between NorA and MdfA. Indeed, even though the identity similarity percentage predicted by Phyre2 between the crystal structure of MdfA (PDB ID 4ZP0) and the NorA model was very low (11% sequence identity), the associated confidence score, obtained by alignment of the sequence, was equal to 100%. Phylogenetically NorA and MdfA are strictly related: they belong in fact to the same transporter superfamily, MFS. Moreover, they also belong to the subfamily of drug/H^+^ antiporters. Thus, with a high degree of confidence, the software considered these two transporters as analogs, therefore hypothesizing a possible conserved transport mechanism. Since the two structures were closely phylogenetically linked, and therefore perform the same function, it is assumed that the folding and the generated structure can be reliable.

### 2.3. Biological Assay of CLM on NorA

While it is well known that CLM is a substrate of MdfA, there is no information in the literature about the possible role of CLM as a NorA substrate. As mentioned before, the superimposition between the generated NorA homology model and 4ZOW (MdfA co-crystallized with CLM) suggested a very close structure organization (RMSD of 1.7 Å). In 4ZOW structure, CLM performed two key interactions with Asn33 and Asp34. However, the visual inspection of the NorA amino acids corresponding to these two MdfA acidic residues highlighted the presence of Ile19 and Gly20 (Supplementary Materials, Figure S3).

Thus, we supposed that NorA could not extrude CLM. In order to have some experimental evidence on this topic, we evaluated the CLM minimum inhibitory concentration (MIC) on two different *S. aureus* strains, one of which was wild-type (SA-1199-norA wt) and the other one overexpressing the norA gene and also possessing an A116E GrlA substitution (SA-1199B-norA+), which is a known fluoroquinolones target [30]. The obtained results showed that CLM had the same MIC values (4µg/mL) against the two used strains, thus highlighting that this compound could retain its antibacterial effect regardless of the NorA efflux pump overexpression. Indeed, MIC values of CPX and EtBr, known NorA substrates, appeared significantly different against SA-1199 and SA-1199B (Table 1). This data clearly demonstrated that CLM is not a NorA substrate.

### 2.4. Refinement of the NorA Predicted Model Using MD

**Figure 1:**
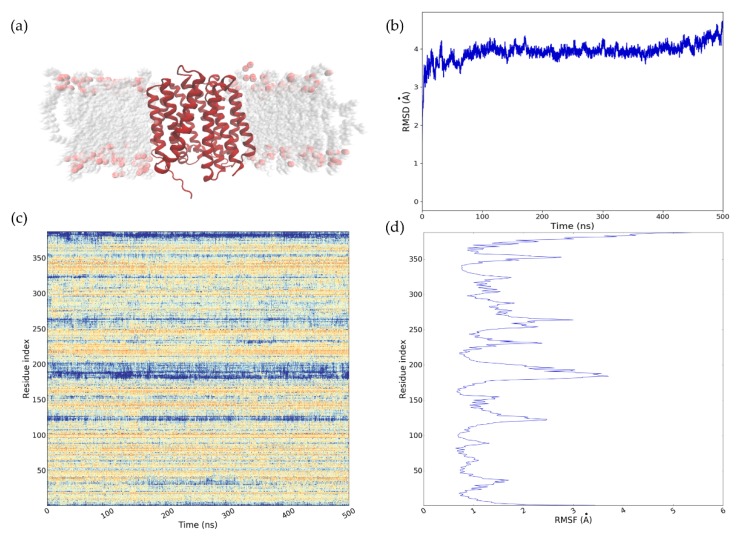
(**a**) NorA homology model embedded in POPC bilayer. (**b**) Calculated RMSD graph of 500 ns of MD simulation of multidrug resistance *S. aureus* NorA efflux pump. Time (ns) is plotted on the x-axis and RMSD (Å) on the y-axis. (**c**) RMSF fluctuation during the MD simulation time of the NorA model. Depending on the intensity of the fluctuation, the color ranges from yellow (low RMSF) to blue, for higher values. (**d**) RMSF of the protein residues.

The chosen homology model (i.e., I-TASSER C_in_) was embedded in a 1-palmitoyl-2-oleyl-glycerol-3-phospho-choline (POPC) bilayer (Figure 1a) and subjected to MD simulations of 500 ns for structural refinement. All the subsequent analyses performed have been conducted in parallel using three different systems, i.e., (i) NorA homology model, (ii) MdfA in complex with CLM (PDB ID 4ZOW) and (iii) MdfA apo. The latter system was used as a reference structure. As highlighted by Figure 1b, the RMSD value of Cα showed good model stability for NorA. RMSD quickly reached a maximum value of approximately 4 Å, which remained steady and constant during the dynamic simulation time. Since the analyzed structure was a homology model, the value obtained, and in particular, the stability achieved can be considered good enough to validate the model. In addition, comparing the RMSDs trends for NorA and MdfA (Figure S4, Supplementary Materials), it was remarkable that the generated homology model became stable after 60 ns of the simulation time and seemed even more stable than the MdfA crystallographic structure. In accordance with the interval time within which SuMD samples binding events, the model can be considered stable.

The most significant residue fluctuations occurred at the level of the loop connecting helix 6 and helix 7 and of the C-term and N-term domains (Figure 1c,d).

Furthermore, to evaluate possible conformational changes during the NorA MD simulation, we clustered the MD conformations using the density-based algorithm DBSCAN [32]. Although the whole MD protein conformations during the trajectory could be divided into two main clusters, we considered only the first cluster for its higher density. Indeed, the first cluster was populated by 4928 protein conformations out of a total of 5000. The centroid conformation of this cluster was then selected for the structural analysis.

The available biological data showed that CPX and CLM were endowed with different specificity for the MdfA and NorA efflux pumps. In particular, while CPX was a substrate of both proteins [30,33], no substrate activity against NorA was observed in our assays for CLM.

Thus, we planned differently in silico approaches (including SuMD simulations) to get insights about the different behavior of the two ligands on MdfA and NorA efflux pumps.

### 2.5. Binding Site Definition and Preliminary Docking

Since the SuMD approach requires the binding site knowledge to address the ligand in the right direction, we performed preliminary docking study only to assess the ability of the two ligands to be hosted into a specific pocket of MdfA and NorA. In a first analysis, we observed whether the crystallographic binding site was translatable into NorA (Figure S5). However, as we had no crystallographic information on NorA, we decided to explore further sites within the pump. Indeed, while for MdfA the binding site was defined by some of the twelve residues that showed interactions to the ligand with the crystallographic ligand (Tyr30, Asn33, Asp34 and 236), to identify the NorA putative binding site, the cluster centroid belonging to the most populated conformation was submitted to SiteMap tool [34] in Maestro suite. The highest-ranked binding site (SiteScore = 1.119) was selected as putative NorA binding site and in particular Ile23, Pro24, Pro27, Tyr225, Ser226, and Gly348 were set as binding site residues. Some of the selected binding site residues are in agreement with some of those residues chosen in previous studies [35]. This site was located more outwards than the CLM binding site. First, three different docking programs (i.e., Glide [36], PLANTS [37] and GOLD [38]) were explored with the aim to identify the best performing method in reproducing the crystallographic binding mode of CLM into MdfA (Supplementary Materials, Table S2). Glide turned out to be the best protocol in generating the correct CLM pose on the basis of the obtained RMSD and E_rvdw (i.e., van der Waals interaction energy) values calculated for each pose. Second, the same Glide protocol was applied to dock CPX against the experimental MdfA pocket, and both CLM and CPX against the hypothesized NorA binding site. The gained results suggested that the two compounds could potentially be hosted in the defined efflux pump binding sites.

### 2.6. Substrate Binding Simulations Using SuMD

#### 2.6.1. General Overview of SuMD Analysis

As already anticipated, in this work the SuMD approach has been applied to MdfA and NorA proteins. Depending on the substrate (CLM or CPX) and on the protein (MdfA or NorA) used in the experiments, four complexes divided into two subsets (A and B) have been subjected to SuMD simulations (as summarized in Table 2). Using the binding site residues previously highlighted, different SuMD simulations were planned. The preliminary docking results suggested that CLM and CPX were potentially able to fit the cavity of the analyzed proteins (i.e., MdfA and NorA).

SuMD replicas of the two studied systems (i.e., *S. aureus* NorA and *E. coli* MdfA) provided some interesting information about the molecular recognition mechanisms and the kinetic processes underlying the interaction between these efflux pumps and their substrates.

First, a self-recognition SuMD simulation of the CLM into MdfA was performed to validate the applicability of the in silico technique. Indeed, this work represents the first example of SuMD applied to efflux pumps. In total, four replicas were performed, and in three of them, CML was able to reach the defined orthosteric site. We refer to these replicas as productive replicas. It is worth noting that in one of the three productive replicas, this approach was able to reproduce the crystallographic binding mode of CLM. Indeed, the RMSD between the experimental and the SuMD pose of CLM was 1.77 Å, underling that the used technique worked pretty well in identifying the correct CLM pose on MdfA, also considering that the crystallographic resolution is 2.4 Å.

The analysis of the SuMD results for CLM on NorA protein showed that a binding event was observed in two replicas. However, the in silico results were not supported by the previously obtained biological assays, which showed that CLM was not a substrate of NorA efflux pump. However, it should be noted that the simulation data only indicated that CLM could be able to enter and reach the binding site (Figure S6). Thus, the fact that geometrically and energetically CLM could be hosted inside NorA did not mean that it had to be extruded at all. For instance, NorA binding by CLM could be compatible with the inhibitory activity of this compound, but unfortunately, no information is available in the literature about this topic to validate or not the hypothesis. The data obtained left the way open for this scenario.

Second, we focused our attention on the recognition of CPX on MdfA and NorA. Both for CPX on MdfA and NorA, the SuMD simulation needed a remarkable number of SuMD steps. In the case of CPX on MdfA to sample a binding event within the orthosteric pocket, we had to increase the number of tries that the system could do before reaching the binding site. This behavior could be explained, considering that during the path the ligand was able to reach a meta-binding state, which was characterized by lower energy compared to that of the final state (Figure S7).

When analyzing the NorA case study results, we observed this behavior again; indeed, in one productive replica, the final state reached by CPX at the defined site was energetically less stable than the ligand pose found at the meta-binding site identified during the path. Moreover, the channel of the pump is rich in charged amino acids. Overall, these observations once more suggested that the kinetic process that allows the substrate to reach the binding is hard and harsh.

#### 2.6.2. SuMD Validation: MdfA-CLM Recognition Pathway

**Figure 2:**
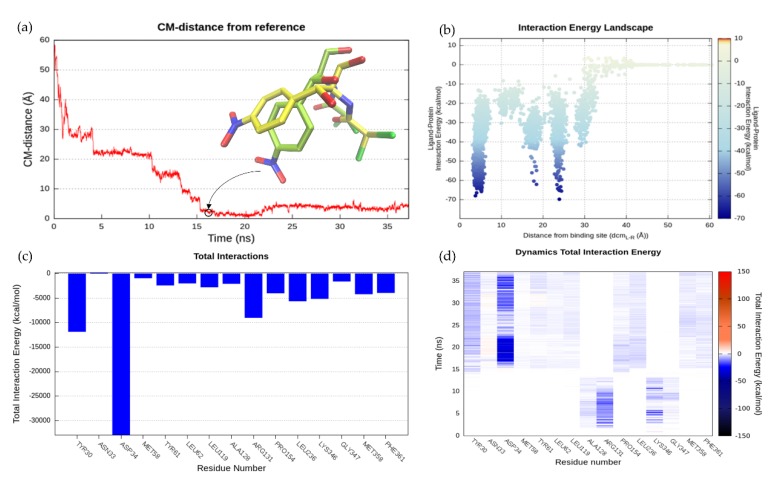
SuMD MdfA-CLM recognition pathway analysis. (**a**) CM-distance between the ligand and the reference binding site calculated as RMSD of simulated position (light green) against the experimental (i.e., crystallographic) one (yellow). (**b**) Interaction Energy Landscape. (**c**) Total Interaction energy plot. (**d**) Dynamics Total Interaction Energy for each ligand-interacting residue.

The CLM was at first positioned 62 Å far away from the MdfA experimental canonical binding site defined by four residues (Tyr30, Asn33, Asp34, and Leu236) (d_cm (L-R)_ = 62 Å). The whole recognition pathway can also be appreciated in this case by browsing Movie S1. The centers of mass distance (d_cm (L-R)_) quickly decreased from the initial 62 Å to about 30 Å during the first 2 ns of the SuMD simulation, as shown in the Dynamic Total Interaction Energy plot (Figure 2d). At this point, CLM established the first contacts with the protein by the “electrostatic recruiters” Arg131 (TM4) and Lys346 (TM10) located at the protein entry. Subsequently, the ligand was stabilized between the two residual recruiters and its center of mass was located at about 20 Å away from the orthosteric site. This recognition mechanism was clearly evident in the Interaction Energy Landscape (Figure 2b) in which there was the first region of minimum; the energy dropped from −40 kcal/mol to −70 kcal/mol. The substrate remained in this position for about 10 ns. Arg131 turned to have a key role in the molecular recognition mechanism, contributing to the binding events with cumulative energy of around −10,000 kcal/mol (Figure 2c). Later, the interaction between CLM and the two mentioned residues stopped and the ligand moved again along the trajectory pathway to penetrate the transporter. The protein region involved in this prolonged interaction could be defined as a meta-binding binding site, as revealed by the stability of MMGBSA energy values (see Movie S1). A meta-binding site is a sort of stopover with enough residence time, which breaks the progressive and continual approach of the ligand. At this point, CLM orientation changed and reached a deeper position inside the canonical binding site, through a horizontal placement, where it makes contacts first with Tyr30 at 16 ns and then with Asp34. Noteworthy, this latter residue showed strong participated in the stabilization of CLM into the canonical binding site by interacting with the substrate OH groups. During the SuMD simulation, CLM was able to reach the orthosteric site in a conformation very close to the crystallographic one, as reported in Figure 2a, where the RMSD reached a minimum value of 1.77 Å at 17 ns. The geometric reproduction of the binding mode can also be observed from Movie S2. The predominant energy role of the amino acids mentioned above can be better understood by looking at the graph of the Total Interactions Energy (Figure 2c). Indeed, the cumulative interaction energy between residue Asp34 and the two ligand oxygens reached the value of −30,000 kcal/mol. Although this residue established contacts with the ligand until the end of the simulation at 37 ns, the substrate changed its binding mode during the interaction time.

To identify the possible CLM recognition sites during the SuMD trajectory, we performed a clustering analysis using DBSCAN (Figure S8). DBSCAN algorithm enables to identify clusters of ligand conformations during the SuMD trajectory, highlighting which regions were most explored by the ligand. Each sphere represents a population of ligand clustered conformations and the sphere radius in relation to the cluster population is set. According to what can be deduced from the analysis carried out, the CLM seemed to have a fairly immediate recognition pathway. Indeed, the clustering analysis identified two main steps characterizing the recognition process: the electrostatic recruitment by the vestibular region residues (Arg131 and Lys346), followed by a rapid transition to the crystallographic binding site, where the higher conformation cluster was identified and retained until the end of the simulation.

#### 2.6.3. NorA-CPX Recognition Pathway

**Figure 3:**
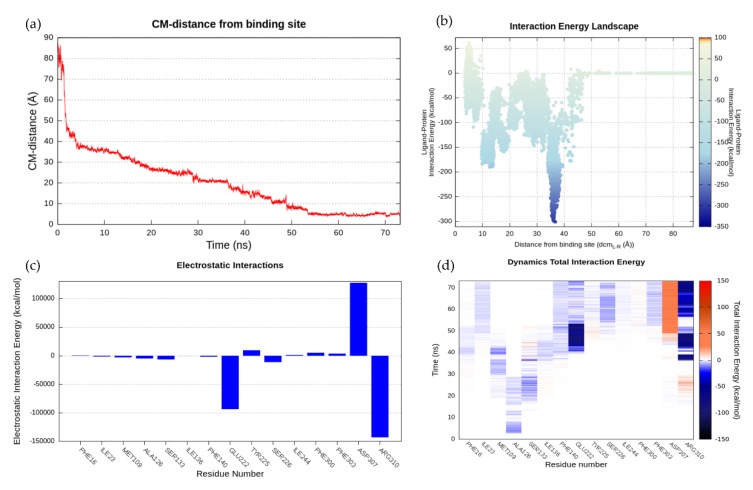
SuMD NorA-CPX recognition pathway analysis. (**a**) CM-distance between the ligand and the binding site. (**b**) Interaction Energy Landscape. (**c**) Total Interaction energy plot. (**d**) Dynamics Total Interaction Energy for each ligand-interacting residue.

**Figure 4:**
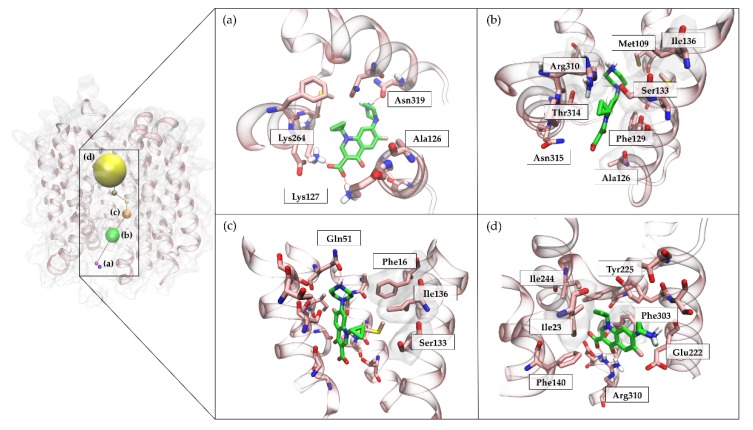
Clustering analysis of CPX recognition pathway during a SuMD trajectory. (**a**) CPX binding mode in the first recognition site. The ligand establishes interactions with Ala126, Lys127, Lys264 and Asn319. (**b**) Panel b shows the interaction between CPX and NorA protein during its trajectory. CPX interacts with Met109, Ala126, Phe129, Ser133, Ile136, Arg310, Thr314, Asn315. (**c**) In cluster c, the ligand interacts with Phe16, Gln51; a hydrophobic contribute comes from Ser133 and Ile136 residues. (**d**) CPX is hosted in the orthosteric binding site. This is also the most populated cluster. CPX mostly establish contacts with Ile23, Phe140, Glu222, Tyr225, Ile244, Phe 303, Arg310.

In the starting geometry, the ligand was placed at a distance of 84 Å far away from the postulated canonical binding site. As depicted in Figure 3d and shown in Movie S3, the first interaction between the ligand and the protein occurred after 3 ns of productive trajectory, involving the Lys127 side chain. The distance between the ligand and protein centers of mass then rapidly decreased from 84 Å to about 40 Å (Figure 3a). This region of first recognition was very rich in positively and negatively charged residues that slowed down the entry of the ligand such as Lys127 Lys264, Glu385, and Asn319 (Figure 3 and Figure 4). This behavior was expected, considering the CPX zwitterionic nature. Indeed, the compound was almost always stabilized in the pump vestibular region by the Lys127 side chain that had strong interactions with the carboxylate group of CPX. As Figure 3a shows, the ligand persisted in this first recognition site until 13 ns. The residence time of the ligand in this region was also supported by the energy interaction of the ligand-protein complex, which reached a value of −300 kcal/mol when the distance between the two centers of mass was between 30 Å and 40 Å (Figure 3b). Therefore, this region was considered a meta-binding site, a key region for the passage of the ligand inside the protein. Subsequently, CPX shifted deeper into the protein by losing the interaction with Lys127, but maintaining the interaction with Asn319. After about 15 ns, the carbonyl group of CPX established again an H-bond with Lys127, whereas the carboxylic group acquired interaction with Tyr131. This binding mode was also stabilized by Ser318. In this second site, the CPX binding mode changed. Indeed, while previously the protonated amine group was located towards the cytoplasmic side, it was now oriented towards the inner periplasmic side of the protein. The ligand was here stabilized by Ser318 and the hydrophobic component had a role in the orientation exploited by Met109. This was another site explored by the ligand, although at a low energy level of −150 kcal/mol (Figure 3b). The arene-H interaction with Thr314 also contributed to the CPX orientation, and this contact was retained until about 30 ns when the distance between the two mass centers was 20 Å. At 30 ns, the ligand again changed its conformation, establishing interaction with Gln51 at the level of the protonated amine. This binding pose was preserved up to about 36 ns, after which CPX began to interact with Arg310. The substrate carboxylic group was engaged in contacts with Arg310, Ser133, and Asn137, while the protonated piperidine nitrogen interacted with Gln51. As can be observed by the IE landscape (Figure 3b) this ligand conformation occurred at about 18 Å of distance from the binding site and was characterized by the energy of −150 kcal/mol. This kind of interactions was retained until 38 ns. Then Gln51 interacted with the carboxylic group of CPX. At about 10 Å from the orthosteric pocket, its orientation was strongly stabilized by Arg310 and Glu222 until 49 ns. The relevance of these two residues was also supported by the histogram of Total Interactions Energy (Figure 3c) and by the Dynamics Total Interaction Energy (Figure 4d). Indeed, Glu222 and Arg310 had total interaction energies of −100,000 kcal/mol and −150,000 kcal/mol, respectively. In addition, this conformation was stabilized by the π-π stacking interaction with Phe140. Finally, CPX shifted to the orthosteric binding site losing the interaction with Arg310 at around 52 ns. The CPX established π-π stacking interaction with Phe303. This orientation was stabilized by Arg310 interaction and the arene-H interaction between the cyclopropyl and the aromatic moiety of the Tyr225. The minimum value of distance observed was 3.6 Å and this conformation persists until the end of the SuMD simulation at 73 ns.

To reveal the most crucial binding sites, a clustering analysis was performed (Movie S4). As Figure 3a shows, two meta-binding sites were identified at about 35 Å distance. As we previously highlighted, several charge residues hosted in this site. Subsequently, the ligand shifted at a distance of about 27 Å from the center of mass of the protein binding site. At this level, we found a third populated site formed by 940 conformations, where the ligand was stationed for a fairly long time (Figure 4a). CPX presented a conformation with the carboxylic group faced towards the cytoplasmic side, while the protonated amine group was directed towards the pump channel all the time. This cluster of conformations was stabilized by Ser318 whose side chain was hydrogen bonded to the CPX carboxylate, and by Met109, Thr314, Ile136, Ser133, Arg310, Phe129, Ala126, which contributed with the van der Waals component (Figure 4b). A further cluster of 653 conformations was found at 18 Å from the binding site cavity, as shown in Figure 4c. Here, the CPX binding mode is characterized by the interaction with Gln51. A small relatively sparsely populated cluster (233 conformations) was identified immediately above the previous one, where this time CPX made polar contact with Gln51 at the level of its carboxyl group. The next cluster identified was that represented in Figure 4d populated by 348 conformations. In this pose, the substrate was firmly stabilized by the two charged residues, Arg310 and Glu222. The CPX final state, identified by the fourth group of conformations, was broadly explored and widely populated (1980 conformations) (Figure 4d).

## 3. Materials and Methods

### 3.1. General

All simulations were performed on a hybrid CPU/GPU cluster. MD and SuMD simulations were carried out with the ACEMD [39] engine on a GPU cluster provided of 18 NVIDIA graphics cards, whose models include GTX 780 to Titan V. Before running MD and SuMD simulations, the following preliminary phases were carried out: (i) protein modeling, (ii) protein-ligand system preparation, (iii) ligand parametrization, and (iv) solvated system setup and equilibration. The protocol based on the CHARMM36/CHARMM general force field (CGenFF) force fields combinations was adopted for transmembrane systems.

### 3.2. Protein Modeling: Preparation of the NorA Target

Quinolone resistance protein NorA amino acid sequence was downloaded in the FASTA format from the UniProtKB database (Uniprot: P0A0J4) [40] and submitted to the different software employed for the 3-D protein structure prediction. Towards this aim, we used I-TASSER [23], SWISS-MODEL [25], RaptorX web server [41] and Phyre2 server [27]. The quality of the NorA 3-D structure models was assessed analyzing the Ramachandran plot generated by MOE suite and QMEANBrane [42]. Model 2 was then refined with MOE Geometry tool. The refined structure was aligned and superimposed on the MdfA crystal structure in the Orientations of Proteins in Membranes (OPM) database [43].

### 3.3. MdfA Crystal Structure Preparation

Protein-ligand complex of *E. coli* was retrieved from the RCSB PDB database (PDB: 4ZOW) [21]. The protein structure to be used as template was prepared with the protein preparation tool as implemented in MOE [29]: hydrogen atoms were added to the complex, and appropriate ionization states were assigned by means of the Protonate-3D tool. Missing atoms in protein side chains were built according to the CHARMM36 force field topology. Missing loops were modeled by the default homology modeling protocol implemented in the MOE protein preparation tool. Non-natural N-terminal and C-terminal domains were capped to mimic the previous residue.

### 3.4. Ligand Preparation

The investigate substrates CLM and CPX are small organic molecules. The substrates were designed using MOE software, after which the partial charges were assigned, followed by a minimization step using the MMFF94 force field. The ligands parameters were achieved from the Paramchem service [44] (CGenFF). Using these initial parameters, we subjected each ligand to 150 ns of preliminary MD simulation. Since the ligands’ behavior observed during the simulation was consistent, we decided to use these parameters for SuMD simulation.

### 3.5. Molecular Docking Experiments

The molecular docking experiments were performed using three different docking protocols: PLANTS [37], GLIDE [36] and GOLD [38]. Starting from the crystal structure, the grid was centered on the center of mass of the co-crystallized ligand (CLM). The grid center was −17.2275, 13.7332 and 24.7736 to x, y and z-axis for all the used protocols. The docking space was defined as a cubic box (22 Å side), with a nested cubic box (10 Å) defining the region where the centroid of the ligand had to be located using Glide. In GOLD and PLANTS protocols, the grid is a sphere with a radius set at 12 Å. Docking on NorA was performed using GLIDE as the best protocol selected. The grid coordinates for NorA model were -28.845, 15.2, 29.82 to x, y and z-axis. Each docking protocol generated 20 poses per ligand. The RMSD and the E_rvdw were calculated with MOE tool.

### 3.6. Solvated System Setup and Equilibration

Four systems composed by the combination of the two analyzed proteins (MdfA and NorA) and the two designed substrates (CLM and CPX) were then prepared. Then, the position of the ligands was manually assigned. To avoid protein-ligand long-range interactions in the starting geometry, CLM and CPX was positioned 62 Å away from the MdfA transporter atom and 84 Å away from the NorA efflux pump atoms, respectively. Transmembrane proteins were embedded in a POPC lipid bilayer, according to the suggested orientation reported in the OPM database. Initial POPC atoms were placed through the VMD membrane builder plugin [45], and lipids within 0.6 Å from amino acid atoms were removed. The membrane used in all the simulations has a dimension of 120Å x 120Å. The systems were solvated with TIP3P water using the program Solvate 1.0 [46] and neutralized by Na+/Cl−counterions to a final concentration of 0.154 M. The systems were then equilibrated through three main steps of molecular dynamics to equilibrate them. In the first stage, after 1500 steps of minimization to allow the system to reduce the clashes between proteins and lipids, 5 ns of MD simulation (2,500,000 steps) were performed in the NPT ensemble, restraining ligand, protein atoms and phosphorousof phospholipid by a positional constraint of 1 kcal mol^−1^ Å^−2^. The temperature was maintained at 310 K using a Langevin thermostat with low damping constant of 1 ps^−1^. The pressure was maintained at 1 atm using a Berendsen barostat; bond lengths involving hydrogen atoms were constrained using the M-SHAKE algorithm with an integration time step of 2 fs. In the second stage, applying the restraints only to the protein and to the ligand and keeping the conditions of constant pressure and temperature (NPT), the temperature was set at 310 K and the pressure at 1 atm, and 10 ns of MD were performed. Then, the last equilibration step included 20 ns of MD simulation and the only restraints left were on the α carbon of amino acids and on the ligand. The stability of the cell volume and POPC area per lipid headgroup during the simulation were evaluated using a script that relies on VMD and GridMAT-MD, a tool for calculating bilayer parameters (Figure S9) [47]. In according with GridMAT-MD values, the area per lipid headgroup ranged from 63 to 70.

### 3.7. Molecular Dynamics (MD) Simulations

MD simulations of 500 ns of the systems (MdfA and NorA C_in_, both without substrates and MdfA in complex with CLM) were performed using ACEMD engine with a time step of 2 fs. The MD trajectory was stridden at 5000 frames. The protein RMSD and RMSF were computed on the protein Cα using VMD trajectory tool. The MD conformations were then clustered using the density-based clustering DBSCAN, setting the RMSD threshold to 2 and the minimum number of protein conformations that could generate a cluster to 30. The cluster centroid was selected using a script based on Numpy [48] and MDTraj python library [49].

### 3.8. Supervised Molecular Dynamics (SuMD)

Each SuMD simulation is composed of a number of consecutive short unbiased MD simulations (600 ps, editable by the user) in which a supervision strategy, based on a tabu search-like strategy, is applied at the end of each simulation. The supervised variable is the distance between the ligand and protein binding site center of mass it is maintained until the protein-ligand distance reaches a preset threshold value (5 Å in this case study). Then the simulation proceeds as a conventional unbiased MD simulation. For a more detailed description of the SuMD analyzer, Salmaso et al. provide all the necessary information [50].

Four simulations were carried out for each system, starting from the same initial geometry is based on the subset. The more significant replicas are described in the results and discussion section.

### 3.9. Analysis of pepSuMD Trajectories

All the trajectories generated by pepSuMD [50] were analyzed by an in-house script written in tcl and python, that makes use of Numpy [48] and ProDy modules [51]. The analyses were then performed on the whole trajectories. In brief, the single SuMD step trajectories were stridden, by a user-defined value (here 10), superposed on the first frame Cα carbon atoms of the target protein, wrapped and merged. The in-house script computes several sides of the SuMD simulation performed. It analyzes the geometry, such as the distance between the ligand and the binding site center of mass and the protein RMSD, the ligand-target interaction energy estimation during the recognition process plotted on the Interaction Energy Landscape plots. This analysis also calculates all the established interactions between the protein and the ligand.

The clustering analysis was performed using the density-based clustering algorithm DBSCAN, setting the RMSD threshold to 1.75 Å and the minimum number of protein conformations that could generate the cluster to 200 for MdfA-CLM and NorA-CPX system.

Representations of the molecular structures were prepared with VMD [45].

### 3.10. Microbiological Assays

The strains of *S. aureus* employed were SA-1199 (wt) and SA-1199B (overexpressing norA and also possessing an A116E Grla mutation). The MIC of the CLM was determined by microdilution technique according to CLSI guidelines [52].

## 4. Conclusions

This work investigated at the molecular level the substrate recognition pathway of NorA through a Supervised Molecular Dynamics (SuMD) approach, using NorA homology models. In this work, different NorA homology models’ structural quality assessment and validation was carried out. These analyses allowed the selection of a NorA model built based on the MSF *E. coli* MdfA transporter, showing an inward conformation with an opening toward the cytoplasmic side as the best starting point for further studies. Notably, the antibiotic CPX is a substrate of both NorA and MdfA efflux pumps, while CLM is a specific substrate of MdfA, as confirmed by our biological experiments.

With this information in hand, a series of SuMD simulations were planned in an attempt to investigate the molecular basis of NorA substrate recognition. To test the ability of the chosen technique in studying these protein systems (i.e., efflux pumps), CLM was used as internal control given that a co-crystal structure between this substrate and MdfA was available.

The obtained results on the MdfA-CLM system supported the choice of the SuMD methodology to study the substrate recognition by efflux pumps. Indeed, CLM was able to reach the orthosteric site in a very close orientation (RMSD of 1.77 Å) with respect to the crystallographic position.

Interesting results were also obtained from the NorA-CPX SuMD simulations. In one of the five replicas, CPX was able to reach the orthosteric site. Additionally, in three replicas, CPX explored a meta-binding state where the strong electrostatic interaction seemed to be critical. This meta-binding site, placed at the interface between the protein and the cytoplasm, could work as the first recognition site for CPX, which was then oriented and released into the protein cavity. During its trajectory, CPX explored several recognition sites, establishing interaction with Lys127, Lys264, Met109, Ser133, Ile136 Gln51, Arg310, Glu222, Phe303, and Tyr225.

To correctly interpret the obtained results, we have to keep in mind that the efflux pumps (e.g., MdfA and NorA) are promiscuous proteins. They are involved in the extrusion of structurally unrelated chemical compounds. Thus, different pathways of substrates recognition can be used on the basis of the specific substrate chemical structure.

The internalization of a molecule into the efflux pump cavity cannot ensure the activation of the protein conformational change required to have the substrate extrusion. Thus, a binding event cannot always correspond to an extrusion event.

In this context, the present work provides a solid homology structural model and an accurate technique (i.e., SuMD) that could rationally aid the comprehension of both the molecular mechanisms of action and inhibition of NorA efflux pump.

## Figures and Tables

**Table 1:** MICs (minimum inhibitory concentration) of EtBr (ethidium bromide), CLM (chloramphenicol), and CPX (ciprofloxacin) in susceptible and resistant *S. aureus* strains.

	SA-1199	SA-1199B
CPX	0.25 ^a^	8 ^a^
CLM	4	4
EtBr	1 ^a^	32 ^a^

^a^ Data from Singh et al. [31].

**Table 2:** SuMD replicas results summary.

System	Replica	Outcome	Time (ns)	Best d_cm (L-R)_ Å
Subset A				
CLM-MdfA	1	productive	32	3.1
CLM-MdfA	2	productive	36	2.9
CLM-MdfA	3	non productive	13	23.4
CLM-MdfA	4	productive	37	3.1
CLM-NorA	1	productive	16	34.4
CLM-NorA	2	productive	58	0.4
CLM-NorA	3	productive	47	1.7
CLM-NorA	4	productive	14	30.7
Subset B				
CPX-MdfA	1	productive	56	3.6
CPX-MdfA	2	non productive	23	16.3
CPX-MdfA	3	productive	44	28
CPX-MdfA	4	productive	47	2.9
CPX-NorA	1	non productive	16	25.5
CPX-NorA	2	non productive	19	26.3
CPX-NorA	3	non productive	26	26.3
CPX-NorA	4	productive	73	3.4

## Supplementary Materials

Supplementary materials can be found at https://www.mdpi.com/1422-0067/20/16/4041/s1. Supplementary videos can be found at https://doi.org/10.5281/zenodo.3370506.

## Abbreviations

MD	Molecular Dynamics
TM	Transmembrane
PDB	Protein Data Bank
C_in_	Inward Conformation
C_out_	Outward Conformation
SuMD	Supervised Molecular Dynamics
MFS	Major Facilitator Superfamily
CPX	Ciprofloxacin
CLM	Chloramphenicol
MIC	minimum inhibitory concentration
EtBr	ethidium bromide
RMSD	Root Mean Square Deviation Standard
RMSF	Root Mean Square Fluctuation Standard
POPC	1-palmitoyl-2-oleyl-glycerol-3-phospho-choline
OPM	Orientation of Proteins in Membrane
NPT	Isothermal-isobaric ensemble
