# Supplementary material for: Deciphering the Molecular Recognition Mechanism of Multidrug Resistance Staphylococcus aureus NorA Efflux Pump Using a Supervised Molecular Dynamics Approach
Source: Int J Mol Sci. 2019 Aug 19;20(16):4041. doi: 10.3390/ijms20164041 (PMC6719125; doi:10.3390/ijms20164041)
Supplement: Supplementary file 1 [file ijms-20-04041-s001.pdf]

International Journal of *Molecular Sciences*

MDPI

Supplementary Materials

Deciphering the molecular recognition mechanism of multidrug resistance Staphylococcus aureus NorA efflux pump using a Supervised Molecular Dynamics approach

Deborah Palazzotti [1,2], Maicol Bissaro 1, Giovanni Bolcato [1], Andrea Astolfi [2], Tommaso Felicetti [2], Stefano Sabatini [2], Mattia Sturlese 1, Violetta Cecchetti [2], Maria Letizia Barreca [2,*] and Stefano Moro [1,*]

Table S1. Summary of the developed NorA homology models.

	**Model 2 I-TASSER**	**Model RaptorX**	***Phyre2***	**Model 1 I-TASSER**	**Model1 Swiss-model**	***Phyre2***
PDB TEMPLATE	4ZOW	4IKV	4ZP0	3WDO	3WDO	3WDO
CONFORMATION	Inward	Inward	Inward	Outward	Outward	Outward
SIMILARITY (%)	28.6	27	27	33.1	31.3	30.7
SEQUENCE IDENTITY (%)	11	16	12	16	16.03	16
RMSD (Å)	1.7	3.23	0.59	0.67	0.47	0.35
RAMACHANDRAN OULIER N°	5	2	10	6	2	5
QMEAN VALUE	-7.06	-7.93	-8.17	-6.76	-5.97	-7.52

Table S2. Results obtained by different docking studies of CLM into MdfA.

	**Glide**		**GOLD**		**PLANTS**	
	RMSD	E_rvdw	RMSD	E_rvdw	RMSD	E_rvdw
pose_1	1.94	-29.63	5.56	-22.40	5.71	-7.55
pose_2	5.54	-29.83	1.52	-20.60	3.16	-11.35
pose_3	1.23	-28.88	1.30	-14.82	5.54	6.49
pose_4	5.50	-29.14	2.41	-25.51	5.70	-8.48
pose_5	5.27	-27.73	5.87	80.99	6.07	-26.20
pose_6	1.53	-28.13	4.63	-15.99	6.08	-26.98
pose_7	2.55	-27.80	2.52	-7.93	2.78	24.99
pose_8	5.39	-27.42	5.75	-22.56	6.11	-18.55
pose_9	5.33	-26.85	5.63	-28.29	2.58	-22.10
pose_10	5.54	-27.36	3.55	-18.53	2.40	5.80

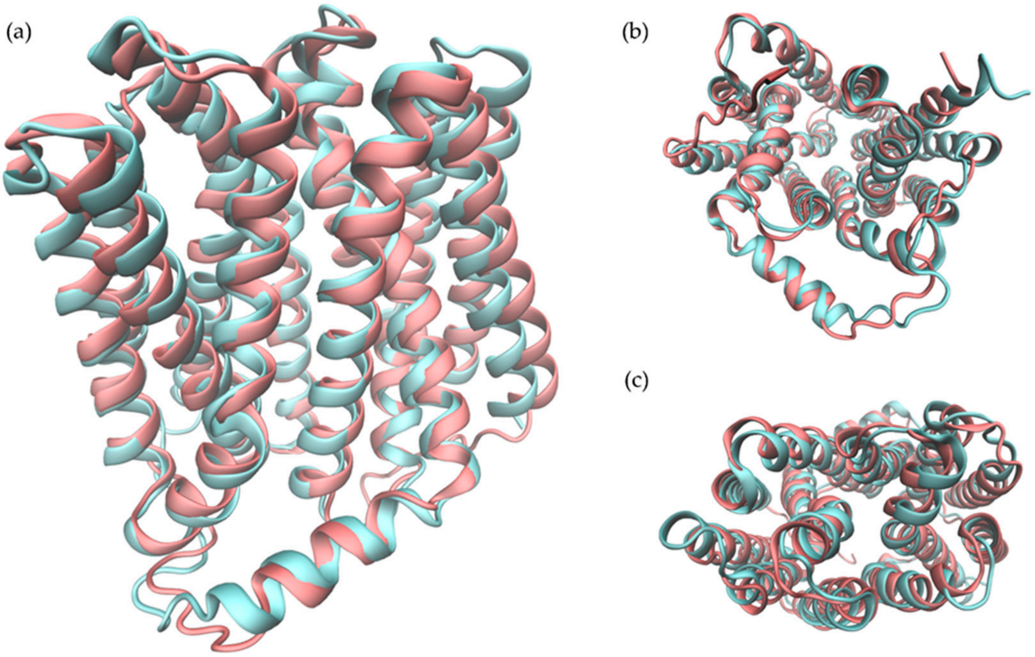

Figure S1. The template MdfA is shown in cyan while NorA in red. Lateral view (a), cytoplasmic side view (b) and periplasmic side view (c).

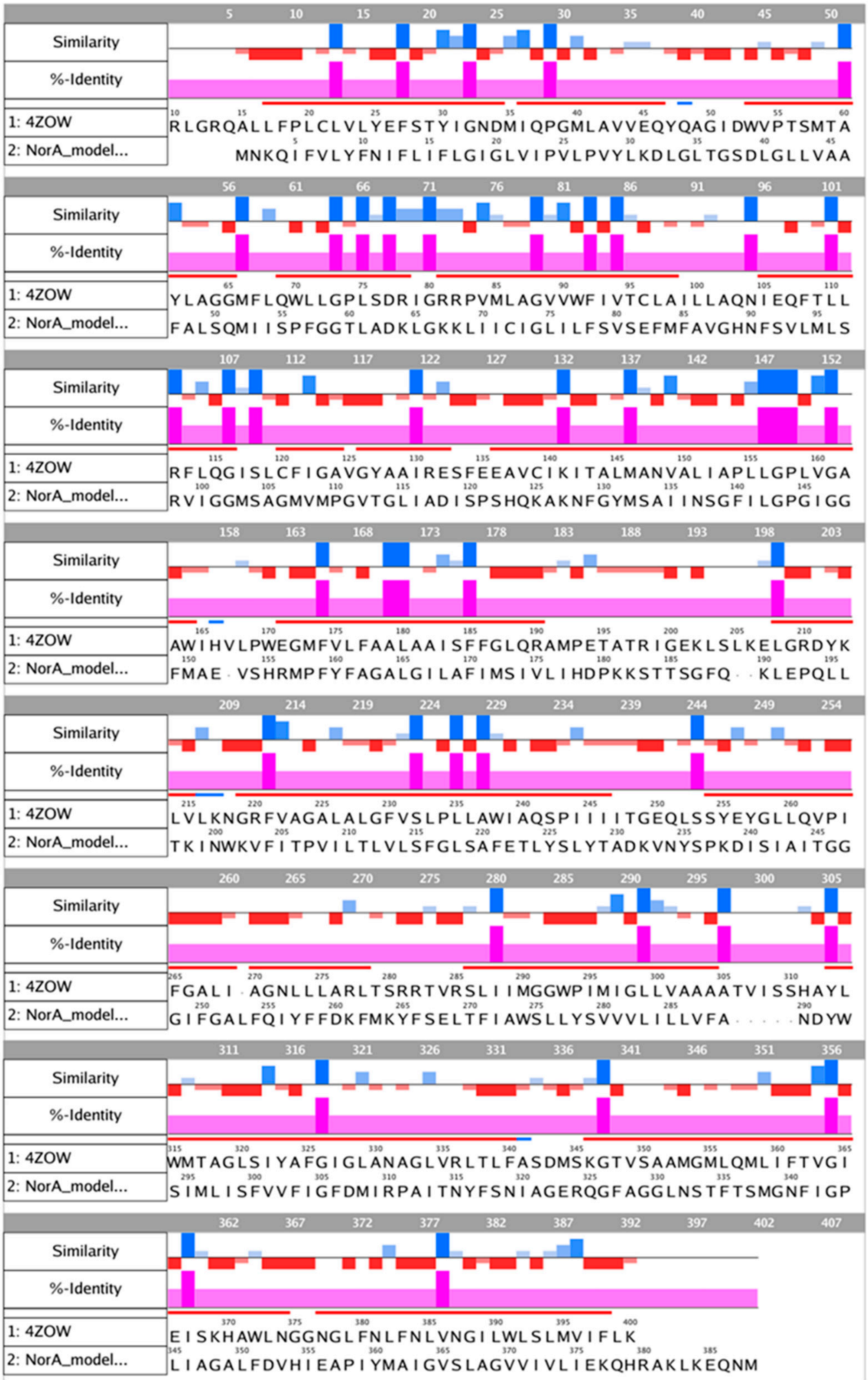

Figure S2. Pairwise alignment for *S. aureus* NorA efflux pump and *E. coli* MdfA sequences, both members of the MFS with 12 TM helices. In pink the sequence identity of the two sequences residues is represented, while in light blue the similarity between the two sequences is depicted. Identical residues are also conserved residues.

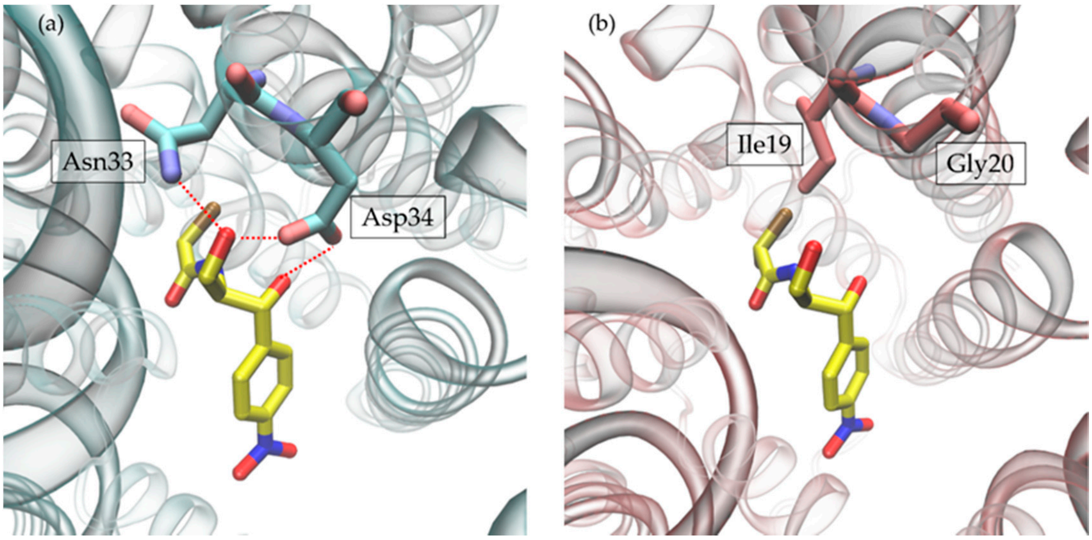

Figure S3. Template and NorA comparison. In Figure (a) the interaction with Asn33 and Asp34 of MdfA with CLM is showed, while in Figure (b) there are no key residues that could establish interactions with the CLM.

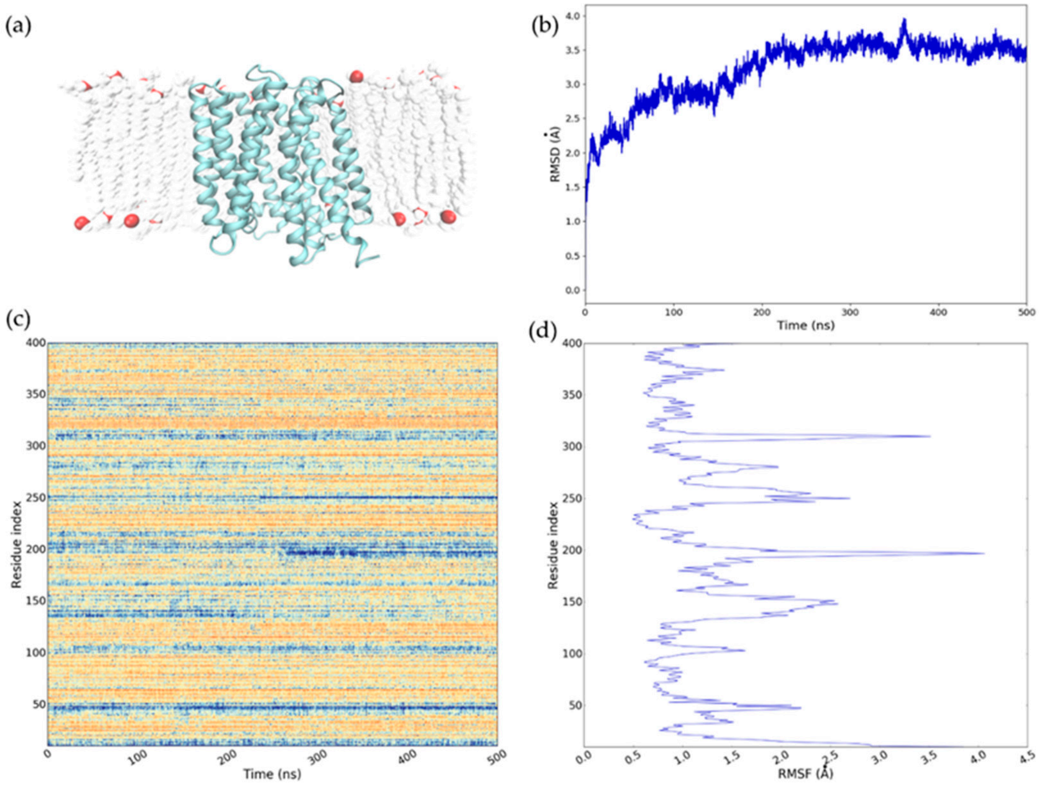

Figure S4. (a) MdfA homology model embedded in POPC bilayer. (b) Calculated RMSD graph of 500 ns of MD simulation of MdfA. Time (ns) is plotted on the x-axis and RMSD (Å) on the y-axis. (c) RMSF fluctuation during the MD simulation time of the MdfA template. Depending on the intensity of the fluctuation, the coloring ranges from yellow (low RMSF) to blue, for higher values. (d) RMSF of the protein residues.

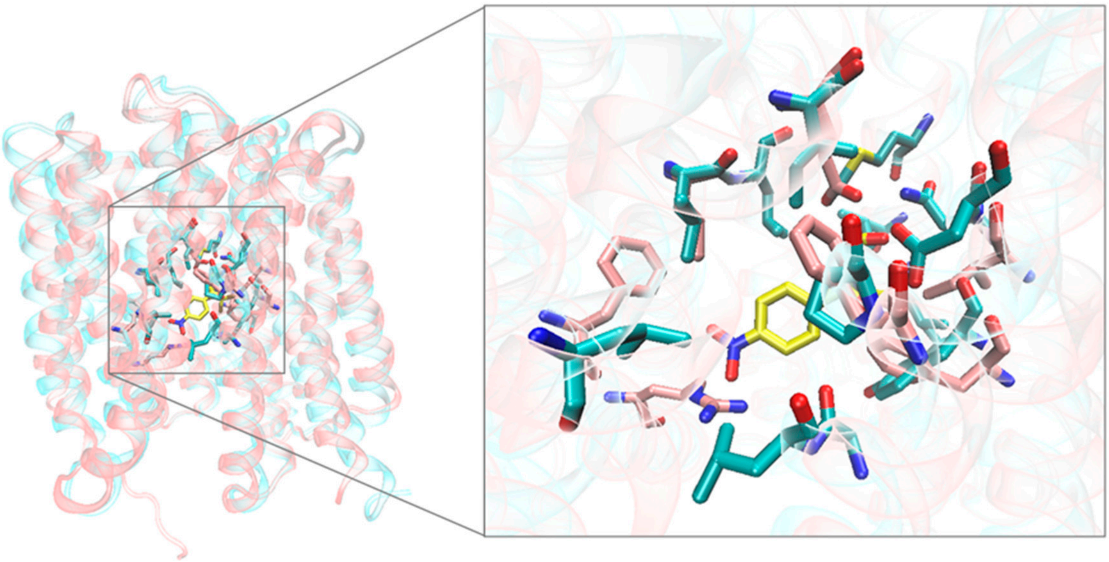

Figure S5. Overlapping MdfA and NorA efflux pump, we could observe that the site of crystallographic recognition is translatable into NorA efflux pump. Here the residues within 3 Å from the ligand are showed. A ligand could be hosted in this NorA binding region.

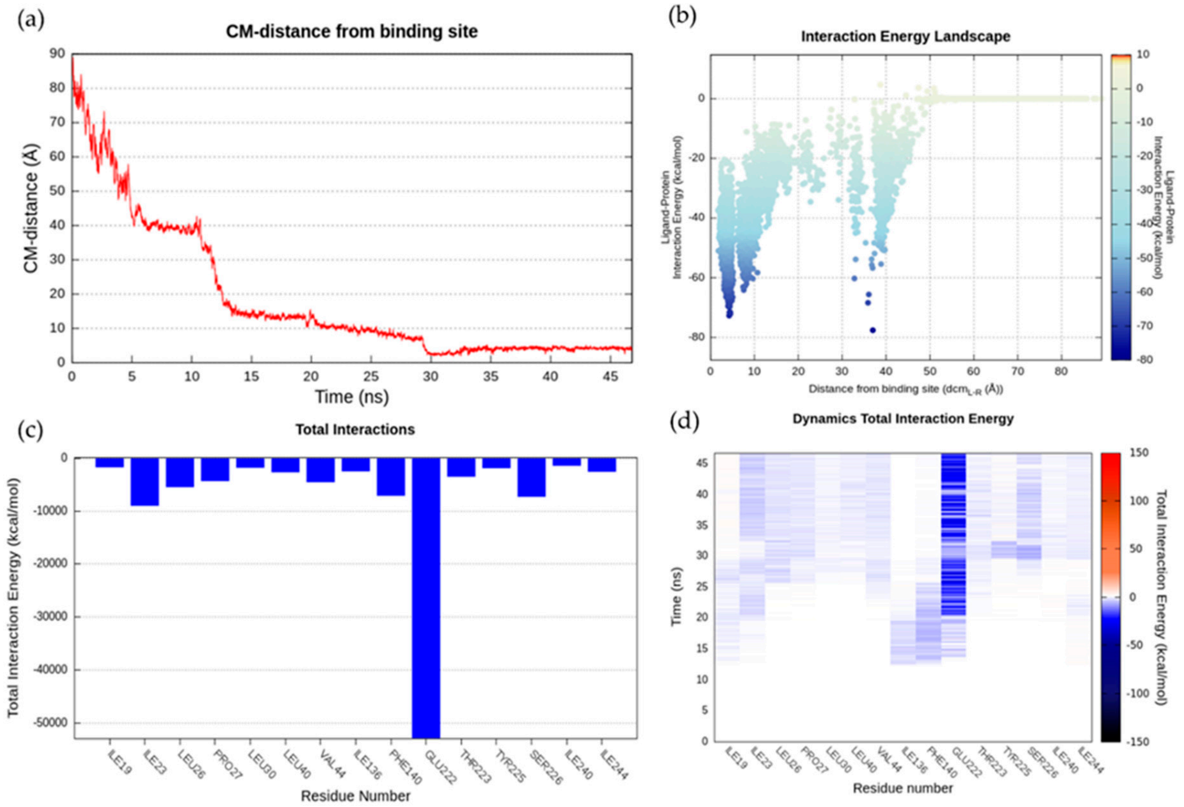

Figure S6. SuMD NorA-CLM recognition pathway analysis. (a) CM-distance between the ligand and the reference binding site. (b) Interaction Energy Landscape(c) Total Interaction energy plot and (d) Dynamics Total Interaction Energy.

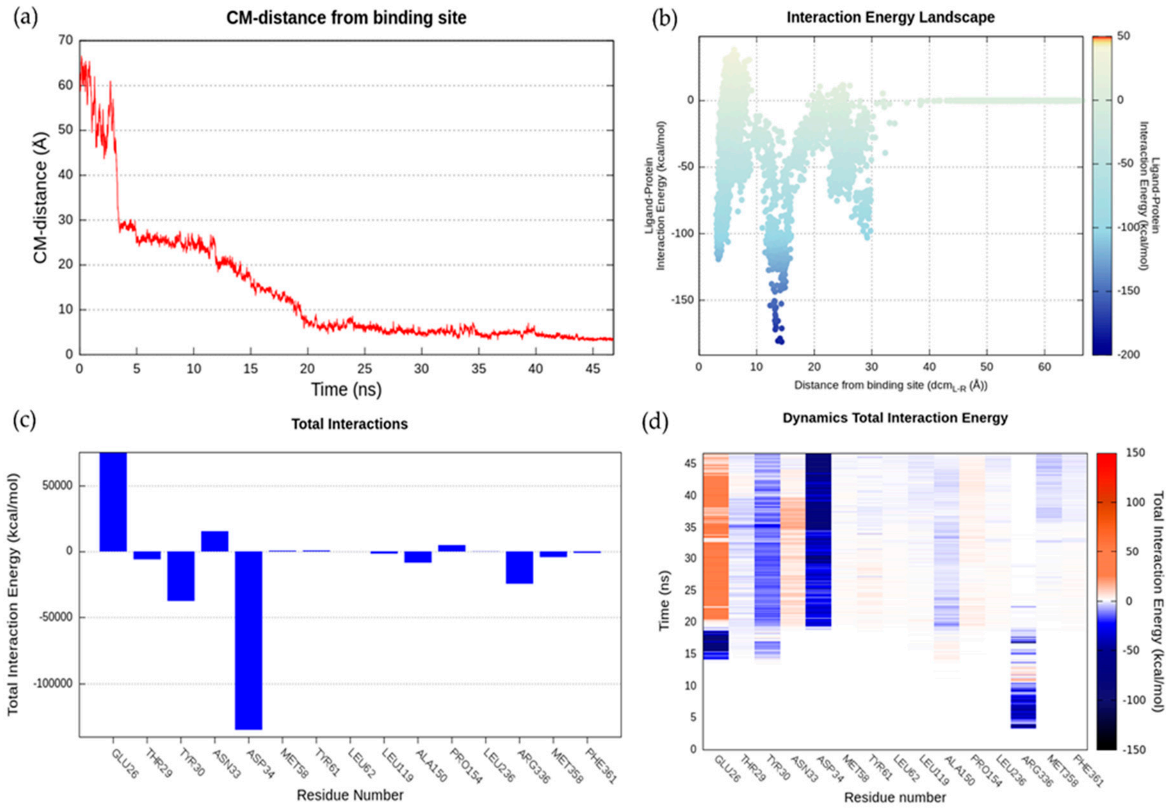

Figure S7. SuMD MdfA-CLM recognition pathway analysis. (a) CM-distance between the ligand and the binding site. (b) Interaction Energy Landscape (c) Total Interaction energy plot and (d) Dynamics Total Interaction Energy.

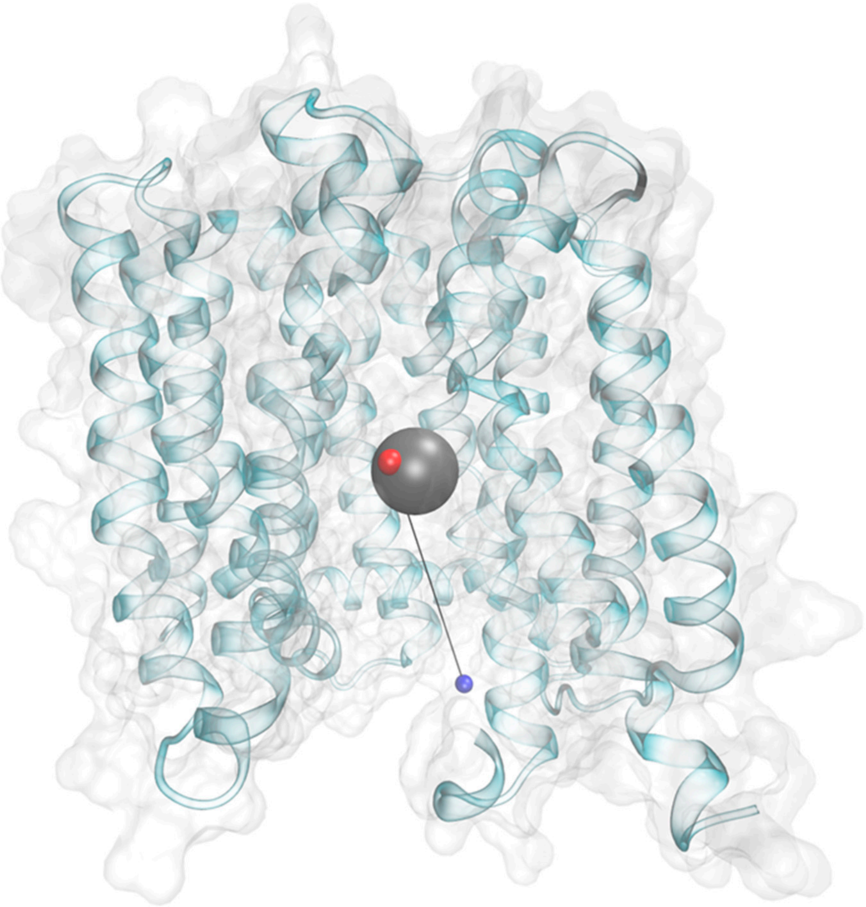

Figure S8. Geometrical clustering of the CLM during the suMD simulation. The MdfA protein is shown in cyan transparency. Here, the protein residues from 140 to 157 are undisplayed. The spheres represent the cluster highlighted. The sphere dimension is in according to the cluster dimension. The first ligand recognition site involves 377 conformations. After this first recognition site, two CLM clusters are depicted in the defined binding site. The first is represented in red and includes 419 while the latter is composed by 1422 conformations and it is represented in grey.

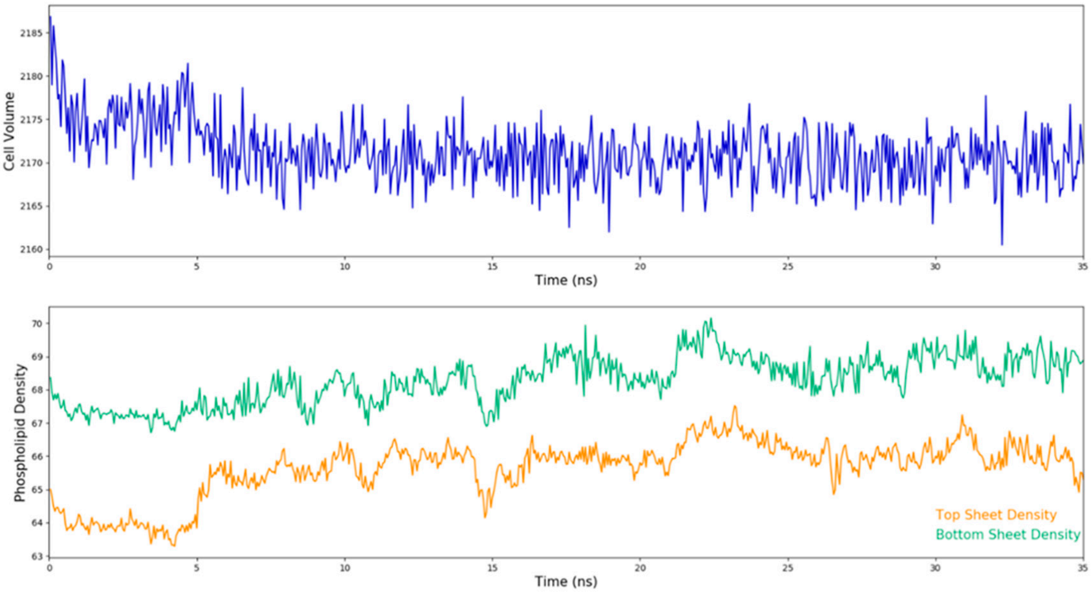

Figure S9. The stability of the modelled NorA system was investigated by using a script written in Perl that relies on VMD and GridMAT-MD [21]. This tool allowed us to observe that, during the equilibration that anticipated the dynamics, the volume of the cell remained constant during the whole balancing phase. In parallel, we also examined fluctuations in lipid bilayer dimensions across the unit cell. We observed that the top and bottom sheet of the lipid bilayer remain stable, according to the range value reported by GridMAT-MD tool. Here we report the volume cell stability (A) and the Phospholipid density (B): the bottom and the top side stability of the lipid bilayer are shown by the green and the yellow line, respectively.

Title of Movie-S1

Movie-S1. MdfA-CLM recognition pathway.

Legend of Movie-S1

The Movie is composed of four synchronized and animated panels that show different aspects of the suMD simulation. The time evolution is reported in a nanosecond. In the first panel (upper left), the molecular representation of the system is shown. The MdfA backbone is represented by the new cartoon style (cyan). The CLM is shown in yellow and by a transparent surface. The protein residues within 3 Å from the ligand are made explicit by a stick representation.

In the second panel (upper-right), the CM-distance between the protein and the ligand centers of mass is reported.

In the third panel (lower left), the MMGBSA energy profile is reported.

In the fourth panel (lower-right) cumulative electrostatic interactions are reported for the 15 MdfA residues most contacted by CLM during the whole simulation.

Title of Movie-S2

Movie-S2. MdfA-CLM recognition pathway.

Legend of Video-S2

The Movie shows the suMD trajectory of CLM on MdfA compared to the CLM crystallographic pose. MdfA is represented in cyan new cartoon transparency. The crystallographic pose is showed in yellow while the experimental one in light green. At 16.69 ns a RMSD value of 1.77 Å is highlighted.

Title of Movie-S3

Movie-S3. NorA-CPX recognition pathway.

Legend of Video-S3

The Movie is composed of four synchronized and animated panels that show different aspects of the suMD simulation. The time evolution is reported in a nanosecond. In the first panel (upper left), the system is shown. The NorA backbone is represented by the new cartoon style (red) and the protein residues within 3 Å of CPX are shown in a stick. CPX is rendered by a green stick.

In the second panel (upper-right), the distance between the center of mass of the ligand and the protein during the trajectory is reported.

In the third panel (lower left), the MMGBSA energy profile is reported. In the fourth panel (lower-right) cumulative electrostatic interactions are reported for the 15 NorA residues most contacted by CPX during the whole suMD trajectory.

It is important to note that the following video has a duration that is half of the simulation of suMD. However, this stride does not alter the description of the trajectory performed by the ligand.

Title of Movie-S4

Movie-S2. Clustering of NorA-CPX recognition pathway.

Legend of Video-S4

The Movie S4 depicts the clustering analysis of CPX during the whole suMD simulation. The NorA protein is shown in red new cartoon transparency. CPX is rendered by a light-green stick and by a transparent surface. The spheres are shown in 7 different colors, according to the different clusters. Each sphere dimension is in according to the cluster dimensions. After a first recognition site, the ligand conformations are clustered in different sites of the NorA channel. It is important to note that the following video has a duration that is half of the simulation of suMD. However, this stride does not alter the description of the trajectory performed by the ligand.
